# Factors Underlying the Early Limb Muscle Weakness in Acute Quadriplegic Myopathy Using an Experimental ICU Porcine Model

**DOI:** 10.1371/journal.pone.0020876

**Published:** 2011-06-14

**Authors:** Julien Ochala, Karsten Ahlbeck, Peter J. Radell, Lars I. Eriksson, Lars Larsson

**Affiliations:** 1 Department of Neuroscience, Uppsala University, Uppsala, Sweden; 2 Section of Anesthesiology and Intensive Care Medicine, Karolinska Institutet, Stockholm, Sweden; 3 Department of Biobehavioral Health, The Pennsylvania State University, University Park, Pennsylvania, United States of America; Medical College of Georgia, United States of America

## Abstract

The basic mechanisms underlying acquired generalized muscle weakness and paralysis in critically ill patients remain poorly understood and may be related to prolonged mechanical ventilation/immobilization (MV) or to other triggering factors such as sepsis, systemic corticosteroid (CS) treatment and administration of neuromuscular blocking agents (NMBA). The present study aims at exploring the relative importance of these factors by using a unique porcine model. Piglets were all exposed to MV together with different combinations of endotoxin-induced sepsis, CS and NMBA for five days. Peroneal motor nerve conduction velocity and amplitude of the compound muscle action potential (CMAP) as well as biceps femoris muscle biopsy specimens were obtained immediately after anesthesia on the first day and at the end of the 5-day experimental period. Results showed that peroneal nerve motor conduction velocity is unaffected whereas the size of the CMAP decreases independently of the type of intervention, in all groups after 5 days. Otherwise, despite a preserved size, muscle fibre specific force (maximum force normalized to cross-sectional area) decreased dramatically for animals exposed to MV in combination with CS or/and sepsis. These results suggest that the rapid declines in CMAP amplitude and in force generation capacity are triggered by independent mechanisms with significant clinical and therapeutic implications.

## Introduction

The severe neuromuscular dysfunction in critically ill intensive care unit (ICU) patients is associated with significant morbidity and mortality. The acquired myopathy, acute quadriplegic myopathy (AQM), seen in critically ill patients is a common cause underlying neuromuscular dysfunction in ICU patients and is characterized by a general paralysis or weakness in all trunk and limb muscles [1], [2]. AQM is associated with a decreased compound muscle action potential (CMAP) amplitude, related to a decreased muscle membrane excitability [3], muscle fibre atrophy and disrupted function of the contractile apparatus related to a general decline in myofibrillar protein content and a preferential partial or complete loss of myosin and myosin-associated proteins [4], [5].

The basic understanding of the mechanisms underlying the impaired muscle function in ICU patients with AQM remains insufficient. Primary disease and sepsis contribute to the weakness, but there is heterogeneity of underlying diseases and pharmacological treatments among patients with similar outcomes. In this context it is likely that several common components of ICU treatment, such as prolonged mechanical ventilation with subsequent immobilization (MV), administration of systemic corticosteroid (CS) and/or neuromuscular blocking agents (NMBA) are involved in the pathogenesis behind this disorder [1], [2], [6]. These factors have all been proposed to be important triggering factors [6], [7], but their relative importance remains unknown. There is therefore a compelling need for an experimental ICU model in which these different factors can be studied separately or in combination. Different experimental animal models have been introduced that have given valuable insights [8], but most of these animal models do not include concurrent ICU conditions such as prolonged mechanical ventilation, sedation, immobilization and sepsis together with common interventions used in modern anesthesiology and intensive care, i.e. systemic CS and NMBA administration [8].

This study aims at unravelling the relative importance of the different factors triggering muscle paralysis in ICU patients. For this purpose, we have used a unique experimental porcine ICU model in an attempt to investigate the relative influence of MV, sepsis, CS and NMBA during the early development of neuromuscular dysfunction, i.e., piglets have been exposed to various combinations of MV, endotoxin-induced sepsis, CS and NMBA for a duration of five days [9], [10], [11]. It was initially hypothesized that: (i) MV and sedation alone is sufficient to decrease CMAP amplitude, muscle fibre size and contractile function; and (ii) sepsis, CS and NMBA together with MV and sedation, separately or in combination, will have a additive negative effect on CMAP amplitude, muscle fibre size and contractile function.

## Methods

### Animals

A detailed description of animals has been given elsewhere [9]. Briefly, 18 female domestic piglets (23–30 kg body weight) were included (Table 1). Animals were sedated with medetomidine (Dormitor vet 1 mg/ml, Orion Pharma AB, Stockholm, Sweden) and zolazepam (Zoletil 250, Reading, Carros, France). After arrival in the laboratory and preparing an iv line, 100 mg of ketamine (Ketaminol vet 50 mg/ml, Intervet, Boxmeer, Netherlands) was administered. During the 5-day study period, the animals were sedated using isoflurane inhalation (Abbott Laboratories, 0.8–1.3% end-tidal concentration) supplemented by intravenous bolus doses of morphine and ketamine as needed. After induction, the trachea was cannulated via a midline incision and a trachestomy tube was placed distally to the cricothyroid cartilage. All animals were then mechanically ventilated using volume-controlled ventilation (Servo 300; Siemens Elema) with an initial FiO_2_ of 0.21–0.30, an inspired tidal volume of 10 ml·kg^−1^ and a respiratory rate of 20 breaths per min, with an I∶E ratio of 1∶2 and inspiratory rise time of 5–10%. During the remaining study period, the settings were adjusted carefully to maintain arterial oxygen and carbon dioxide tensions within normal limits and to avoid high airway pressures and risk of barotrauma. Sedation was titrated to promote ventilator synchrony and inhibit spontaneous breathing activity. Arterial and central venous catheters were placed in the common carotid artery and the internal jugular vein via a separate incision on the neck and under antiseptic conditions. Arterial and central venous blood pressures, airway pressures and volumes as well as dynamic compliance, rectal temperature and standard three-lead ECG were continuously monitored. For assessment of the hemodynamic conditions, a Swan-Ganz standard thermodilution pulmonary artery catheter was inserted through the internal jugular vein catheter and advanced until the characteristic pulmonary artery pressure curve was registered. Excessive heat loss was avoided by covering the animals when necessary with a warming blanket. A urinary catheter was placed in the urinary bladder for continuous monitoring of urinary output. Arterial acid-base balance, oxygen and carbon dioxide tensions, electrolytes and blood glucose concentration were monitored using ABL 2 and OSM2 analyzers (Radiometer) and a Glucometer (Bayer Health Care). A continuous infusion of Ringer's acetate at 2000–4000 ml.day^−1^ was given for fluid replacement together with a continuous infusion of buffered glucose 2.5 mg·ml^−1^ adjusting the infusion rate to maintain tight control of arterial blood glucose levels between 4–8 mM, and to assure a urinary output of 25–50 ml·h^−1^ throughout the study period. Enteral feeding was not considered practical in this model due to the relatively deep sedation, in some cases paralysis and a small laparotomy used in some study protocols. Previous attempts to provide additional calories with parenteral nutrition solutions resulted in fat vacuolization in muscle cells [4]. All wounds were carefully cleaned and closed by standard sutures to avoid unwanted contamination. During the whole study period, from day 2 each animal received prophylactic streptomycin 750 mg·d^−1^ and benzylpenicillin 600 mg·d^−1^ (Streptocillin Vet, Boeringer-Ingelheim). The animals were clinically monitored and cared for continuously for the entire study period by one of the authors or by a nurse anesthetist experienced in the care of piglets and knowledgeable about the study protocol. Routine care included evaluation of sedation level, changing position, suctioning, adjusting fluid administration, etc. After initial preparation, animals were allowed to stabilize for one hour. This study protocol was approved by the Karolinska Institutet Ethical Committee on Animal Research (Permit numbers: Dnr N71/98, N54/02 and N75/04).

**Table 1:** Animal treatment.

Group	N	Mean weight	Survival	PaO2 (kPa)	Compliance (ml/cm H_2_0)	BE (mmol/L)	PP (cm H_2_0)	Endotoxin	CS	NMBA
MV	4	27.10 kg	5 days	18.8	22	3.8	28	-	-	-
Sepsis	4	25.60 kg	5 days	17.2	22	1.8	26	E. coli	-	-
CS	3	26.90 kg	5 days	17.3	26	5.2	23	-	Hydrocortisone	-
NMBA	3	26.00 kg	5 days	21.3	25	3.3	25	-	-	Rocuronium
ALL	4	26.80 kg	5 days	19.8	24	4.3	23	E. coli	Hydrocortisone	Rocuronium

MV (mechanical ventilation), sepsis (endotoxemia was induced by a continuous infusion of Escherichia coli endotoxin), NMBA (neuromuscular blocking agent, i.e., continuous infusion of rocuronium, 25 mg·h^−1^), CS (corticosteroid given as bolus doses of hydrocortisone 50 mg, ×3 daily) and ALL (MV+CS+sepsis+NMBA). N: number of animals; BE: base excess; PP: peak pressure.

### Experiment

The animals were randomly assigned to one of the five groups: MV, sepsis, NMBA, CS, and ALL. MV group refers to piglets that were mechanically ventilated for five days. Sepsis group refers to mechanically ventilated piglets in which endotoxemia was induced by a continuous one-hour infusion of Escherichia coli endotoxin, serotype O26∶B6 (Sigma Labkemi), titrated to effect with a mean total dose of 8 µg·kg^−1^. The infusion was started at a low dose and titrated upward until a hemodynamic response occurred consisting of a fall in arterial mean blood pressure ≥30% from baseline, with an increase ≥50% in pulmonary artery occlusion pressure from baseline. The infusion was paused if the animals required fluid resuscitation or administration of adrenalin for bradykardia and was then restarted at a lower dose. If the animal required repeated interventions the infusion could be terminated earlier than at one hour, and if tolerated could run for up to four hours. Continuous infusions of inotropic drugs were not used to support the circulation. This regimen typically resulted in a 6 to 12 hour-period of severe circulatory instability and oliguria (urinary output <25 ml·h^−1^). Animals surviving this period of septic shock generally regained circulatory stability during the remaining experimental period. NMBA group refers to mechanically ventilated piglets in which a neuromuscular blocking agent was administered as a continuous infusion of rocuronium (Esmeron, Organon) 25 mg·h^−1^ for 5 days while CS group refers to mechanically ventilated piglets in which a corticosteroid was given as bolus doses of 50 mg of hydrocortisone (Solu-Cortef, Pfizer AB) administered three times daily throughout the experiment. ALL group refers to piglets that were mechanically ventilated for 5 days together with the induced-endotoxemia, corticosteroid administration three times daily and continuous systemic administration of a neuromuscular blocking agent. Animals were euthanized by a lethal injection of pentobarbitone to terminate the experiment.

### Electrophysiology

The electroneurography (ENeG) analysis included peroneal motor nerve conduction velocities and facial nerve motor nerve distal latency recordings bilaterally on the first and final day (day 5) of the experiment. The tibialis anterior muscles compound muscle action potential (CMAP) amplitudes were recorded upon supra-maximal stimulation of the motor nerve. The electromyography (EMG) analysis included concentric needle EMG examination of proximal hind limb muscles (m. biceps femoris and gluteus maximus) bilaterally (Keypoint, Dantec/Natus, Skovlunde, Denmark).

### Muscle Biopsies and Permeabilization of Fibres

Muscle biopsies were obtained using the percutaneous conchotome method from the biceps femoris muscle at day 1 and 5 of the experiment for all the piglets. The initial biopsies on day 1 were taken before the start of infusion of endotoxin, CS and/or NMBA. Biopsy specimens were dissected into two parts. One part was frozen in liquid nitrogen-chilled propane and stored at −80°C. The other was placed in relaxing solution at 4°C, and bundles of ∼50 fibres were dissected free and then tied with surgical silk to glass capillary tubes at slightly stretched lengths. The muscle bundles were then treated with skinning solution (relaxing solution containing glycerol; 50∶50 v/v) for 24 hours at 4°C, after which they were transferred to −20°C. The muscle bundles were treated with sucrose, a cryo-protectant, within 1–2 weeks for long-term storage [12]. After the sucrose treatment, muscle bundles were detached from the capillary tubes and snap frozen in liquid nitrogen-chilled propane and stored at −160°C.

### Single Permeabilized Muscle Fibre Experimental Procedure

On the day of an experiment, a fibre segment 1 to 2 mm long was left exposed to the experimental solution between connectors leading to a force transducer (model 400A, Aurora Scientific) and a lever arm system (model 308B, Aurora Scientific) [13]. The total compliance of the attachment system was carefully checked and remained similar for all the single muscle fibres tested (5±0.5% of the fibre length). The apparatus was mounted on the stage of an inverted microscope (model IX70; Olympus). While the fibre segments were in relaxing solution, the sarcomere length was set to 2.65–2.75 µm by adjusting the overall segment length [14]. The diameter of the fibre segment between the connectors was measured through the microscope at a magnification of ×320 with an image analysis system prior to the mechanical experiments. Fibre depth was measured by recording the vertical displacement of the microscope nosepiece while focusing on the top and bottom surfaces of the fibre. The focusing control of the microscope was used as a micrometer. Fibre cross-sectional area (CSA) was calculated from the diameter and depth, assuming an elliptical circumference, and was corrected for the 20% swelling that is known to occur during skinning [13].

Relaxing and activating solutions contained (in mM) 4 Mg-ATP, 1 free Mg^2+^, 20 imidazole, 7 EGTA, 14.5 creatine phosphate, and KCl to adjust the ionic strength to 180 mM. The pH was adjusted to 7.0. The concentrations of free Ca^2+^ were 10^−9^ M (relaxing solution) and 10^−4.5^ M (activating solution), expressed as pCas (i.e., −log [Ca^2+^]). Apparent stability constants for Ca^2+^-EGTA were corrected for temperature (15°C) and ionic strength (180 mM). The computer program of Fabiato [15] was used to calculate the concentrations of each metal, ligand, and metal-ligand complex.

At 15°C, immediately preceding each activation, the fibre was immersed for 10–20 s in a solution with a reduced Ca^2+^-EGTA buffering capacity. This solution is identical to the relaxing solution except that the EGTA concentration is reduced to 0.5 mM, which results in more rapid attainment of steady force during subsequent activation.

#### Force

Force was calculated as the difference between the maximal steady-state isometric force in activating solution and the resting force measured in the same segment while in the relaxing solution. Maximal force production was normalized to CSA and termed specific force.

#### Unloaded shortening velocity

At pCa 4.5, once steady-state isometric force was reached, a series of slacks of various amplitudes were rapidly introduced (within 1–2 ms) at one end of the fibre [16]. Slacks were applied at different amplitudes ranging from 7 to 13% of the fibre length. The fibre was re-extended between releases while relaxed in order to minimize changes in sarcomere length. During the slack test, the time required to take up the imposed release was measured from the onset of the length step to the beginning of the tension redevelopment. A straight line including four or more data points was fitted to a plot of release length versus time, using least-squares regression. The slope of the line divided by the fibre segment length was recorded as the maximum unloaded shortening velocity for that fibre segment (V_0_).

For contractile measurements, strict acceptance criteria were applied. First, the sarcomere length was monitored during the experiments, using a high-speed video analysis system (model 901A HVSL, Aurora Scientific). A muscle fibre was accepted and included in the analyses: (i) if the sarcomere length of a single muscle fibre changed <0.10 µm between relaxation and maximum activation, (ii) if maximal force changed <10% from first to final activation, and if *r* of the slope (plot of release length versus time) for V_0_ calculation was >0.96 [17].

After the mechanical measurements, each fibre was placed in urea buffer (120 g urea, 38 g thiourea, 70 ml H_2_0, 25 g mixed bed resin, 2.89 g dithiothreitol, 1.51 g Trizma base, 7.5 g SDS, 0.004% bromophenol blue) in a plastic micro centrifuge tube and stored at −80°C.

### Myosin Heavy Chain (MyHC) isoform expression

Single muscle fibre MyHC isoform expression was determined by 6% sodium dodecyl sulfatepolyacrylamide gel electrophoresis (SDS-PAGE). Sample loads were kept small (equivalent to ∼0.05 mm of fibre segment) to improve the resolution of the MyHC bands (slow and fast MyHC: type I, IIa, IIx and IIb). Electrophoresis was performed at 120 V for 24 h with a Tris–glycine electrode buffer (pH 8.3) at 15°C (SE 600 vertical slab gel unit, Hoefer Scientific Instruments). The gels were silver-stained and subsequently scanned in a soft laser densitometer (Molecular Dynamics) with a high spatial resolution (50 µm pixel spacing) and 4096 optical density levels.

### MyHC and actin contents

The biceps femoris muscle frozen samples were cut at their greatest girth perpendicular to the longitudinal axis of muscle fibres into 10-µm-thick cross-sections with a cryotome (2800 Frigocut E, Reichert-Jung) at −20°C. Sections were dissolved into 100 µl of urea buffer after centrifugation and heating (90°C for 2 minutes) and a volume of 4 µl was loaded on 12% SDS-PAGE. The total acrylamide and Bis concentrations were 4% (wt/vol) in the stacking gel and 12% in the running gel. The gel matrix included 10% glycerol. Electrophoresis was performed for 5 h with a Tris–glycine electrode buffer (pH 8.3) at 15°C (SE 600 vertical slab gel unit, Hoefer Scientific Instruments). The gels were stained with Coomassie blue (0.5 g brilliant blue, 225 ml MeOH, 225 ml distilled H2O and 50 ml acetic acid), as this staining shows high reproducibility and the ability to penetrate the gel and stain all proteins present, i.e., allowing accurate quantitative protein analyses. The gels were subsequently scanned to determine the MyHC to actin ratio.

### Statistical analysis

Data are presented as means ± standard error of the means (SEMs). Sigma Stat software (Jandel Scientific) was used to generate descriptive statistics. For motor nerve conduction velocities and compound muscle action potential amplitudes, a two-way ANOVA (day×group) was applied followed by the Tukey's test. For single muscle fibre experiments, a total 365 fibres were included in the analyses (minimum of 10 acceptable fibres per animal per day). Given the small number of hybrid (type I/IIa and II/IIx) and pure IIb fibres observed in these experiments, comparisons were restricted to fibres expressing the type I, IIa and IIx MyHC isoforms. For CSA, specific force and V_0_, a three-way ANOVA (day×group×fibre type) was performed followed by the Tukey's test. For the MyHC to actin ratio, another two-way ANOVA (day×group) was carried out. Otherwise, linear regression analysis for slack-test determination and nonlinear regression for relative force-pCa curve fitting were performed and relationships were considered significantly different from zero at p<0.05.

## Results

### Electrophysiology

**Figure 1:**
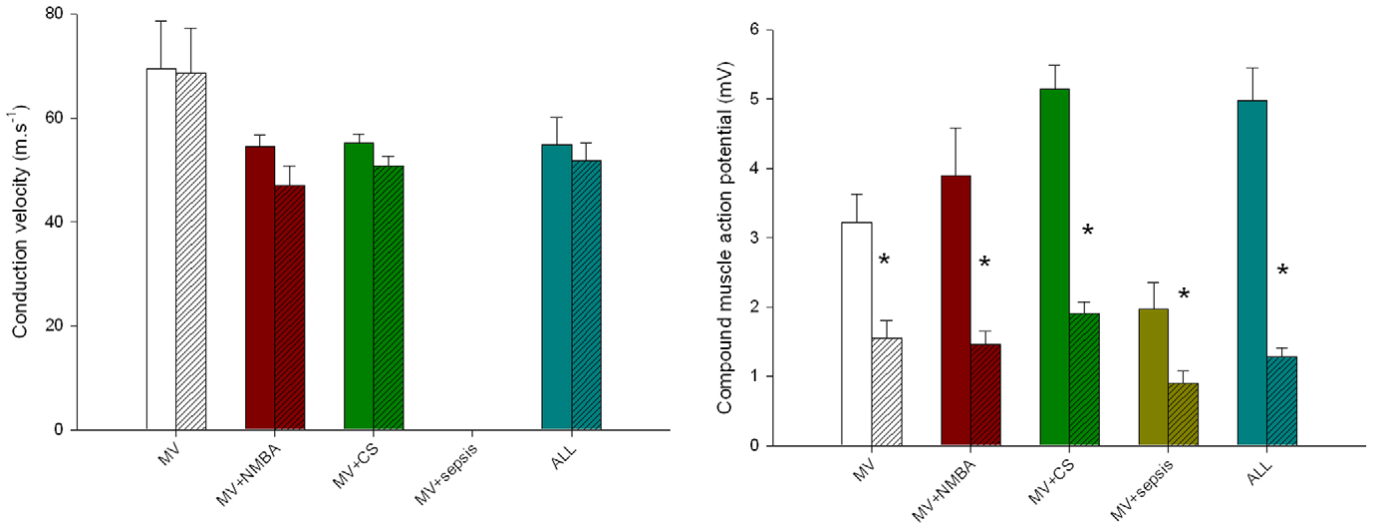
Compound muscle action potential amplitudes. CMAP amplitudes on day 1 and day 5 (normalized to day 1 values) from mechanically ventilated/sedated/immobilized (MV group) piglets, MV together with neuromuscular blocking agents (NMBA group), MV together with corticosteroid administration (CS group), MV together an endotoxin-induced sepsis (sepsis), and MV together with sepsis, CS and NMBA (ALL group).

The average peroneal motor nerve conduction velocity varied between 55 and 69 m·s^−1^ in the different groups on day 1 and between 47 and 69 m·s^−1^ on day 5. No significant change in motor nerve conduction velocity was observed in any of the groups (MV, CS, NMBA and ALL). On the other hand, the CMAP amplitude decreased (p<0.05) significantly after five days in all groups irrespective of the type of intervention (Figure 1). The largest relative decrease in the CMAP amplitude was observed in the all treatment group. However, it should be mentioned that the number of animals in the different groups was relatively small, due to the extremely demanding experimental conditions, and thereby reducing the power of the statistical analyses. Moreover, variability between animals in CMAP amplitude was present. Such variability may be primarily due to individual differences in muscle size and conduction of the EMG signal thorough subcutaneous and skin tissue. Nevertheless, here, CMAP amplitude was compared within the same animals rather than between animals during the 5-day observation period. Therefore, this variability does not have any impact on the conclusions.

### Single muscle fibre size and contractile function

**Figure 2:**
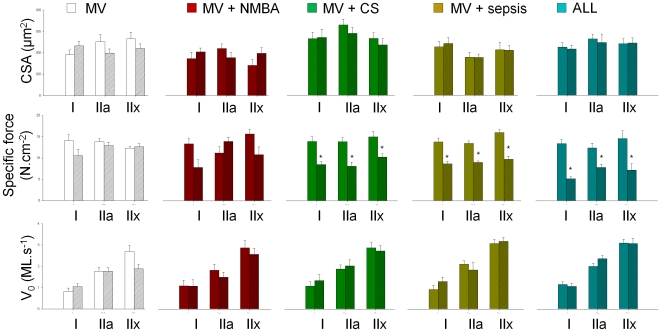
Single muscle fibre size and contractile function. Cross-sectional area (CSA), specific force (maximal force production normalized to CSA) and maximum unloaded shortening velocity (V_0_). Values for day 1 (empty colored bars) and day 5 (hatched colored bars) from mechanically ventilated/sedated/immobilized (MV group) piglets, MV together with neuromuscular blocking agents (NMBA group), MV together with corticosteroid administration (CS group), MV together an endotoxin-induced sepsis (sepsis), and MV together with sepsis, CS and NMBA (ALL group). Data are presented as means ± SEMs. Asterisk denotes a statistically significant difference compared with day 1 (p<0.05).

A total of 365 fibres fulfilled the strict criteria for acceptance (see Materials and Methods) and were included in the analyses. The CSA was unchanged during the five-day period in mechanically ventilated piglets irrespective of the type of intervention (MV, CS, sepsis, NMBA and ALL) (Figure 2). On the other hand, a dramatic decrease in single muscle fibre specific force (maximum force normalized to CSA), irrespective MyHC isoform expression, was observed in animals exposed to CS, sepsis and a combination of CS, sepsis and NMBA (p<0.05) (Figure 2). In single muscle fibres separated according to MyHC isoform expression (types I, IIa and IIx), the maximum unloaded shortening velocity (V_0_) did not change over the five-day period in any of the groups, arguing against a modification of myosin cross-bridge cycling kinetics underlying the decreased specific force in animals exposed to systemic CS administration or sepsis or both (Figure 2). It should be noticed that CSA, specific force and V_0_ were variable between animals at day 1. This variability was not statistically significant. The underlying mechanisms of this variability remain unknown but are probably age-related. In fact, instead of testing adult pigs, piglets were used (cost-related reasons). Maturation and aging are animal-dependent and may potentially modulate CSA, specific force or V_0_.

### Myosin and actin contents

**Figure 3:**
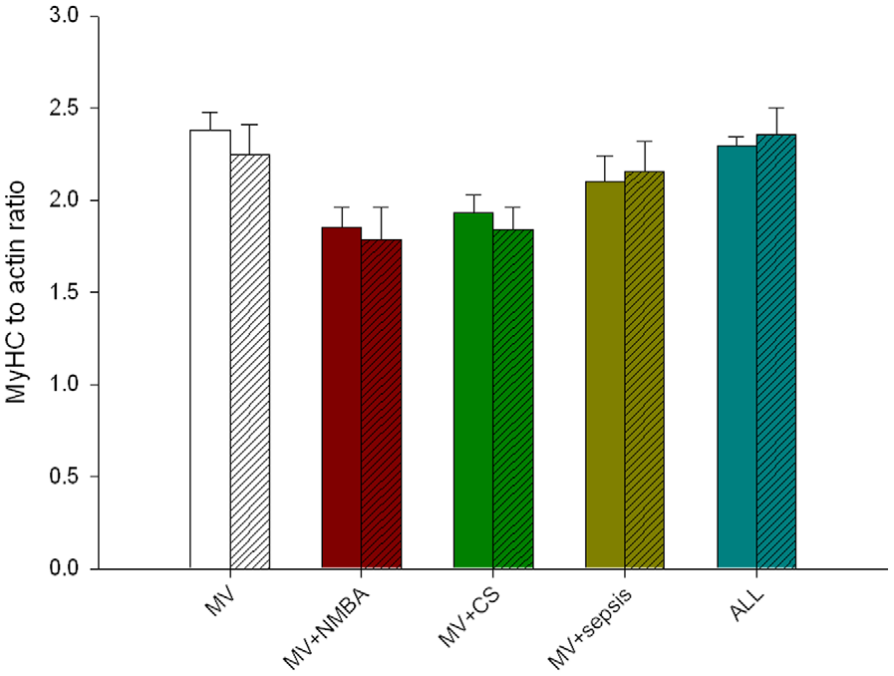
Myosin∶actin ratios. Values for day 1 (empty colored bars) and day 5 (hatched colored bars) from mechanically ventilated/sedated/immobilized (MV group) piglets, MV together with neuromuscular blocking agents (NMBA group), MV together with corticosteroid administration (CS group), MV together an endotoxin-induced sepsis (sepsis), and MV together with sepsis, CS and NMBA (ALL group). Data are presented as means ± SEMs.

There was no preferential myosin loss in any of the groups during the five day period, i.e. myosin∶actin ratios were unaffected in all groups (MV, sepsis, CS, NMBA and ALL groups) (Figure 3).

## Discussion

In a previous pilot study we introduced the porcine ICU model and demonstrated the feasibility of this model [9]. The present study confirms and extends our previous pilot observations in a larger group of animals exposed to different factors suggested as triggers for acute quadriplegic myopathy in ICU patients. These triggering factors (CS, NMBA and sepsis) were given separately and in combination to sedated and mechanically ventilated animals. Results show that MV, sedation and immobilization with or without NMBA, CS and sepsis cause a decreased amplitude of the CMAP. Muscle fibre size, measured in single muscle cell segments at optimal sarcomere length for force generation, was not affected in any of the groups independent of intervention in accordance with our pilot observations [9]. However, a dramatic decrease in force generation capacity (specific force) was observed in animals exposed to CS and sepsis, separately or in combination.

A decreased amplitude of the CMAP, or an absent CMAP, is considered a hallmark of AQM primarily reflecting a decreased muscle membrane excitability [18]. However, other factors may also cause a reduction of the CMAP amplitude, such as muscle atrophy, axonal motor neuron loss, demyelinating neuropathy with conduction block and temporal dispersion of the CMAP or altered recording conditions related to e.g. subcutaneous edema. Muscle atrophy and a demyelinating neuropathy seem highly unlikely due to the maintained muscle fibre size and motor nerve conduction velocity. A motor neuron loss in all groups cannot be completely ruled out, but appears equally unlikely during this relatively short observation period. In septic animals, we have observed increased subcutaneous edema, but the edema was primarily restricted to the craniofacial region and we have not observed clinical signs of edema in the hind-limbs. Although subcutaneous edema cannot be ruled out as a mechanism underlying the reduced CMAP amplitude, an altered membrane excitability appears to be more likely. In a series of elegant experimental studies, Rich and co-workers [19], [20], [21] have presented results showing that the most likely mechanisms underlying the absent or low CMAP amplitudes in patients with AQM are altered membrane excitability and defective sodium channel regulation. Assuming the same mechanism underlying the decreased CMAP amplitude in our porcine ICU model, the present results suggest that inactivity *per se* is a very important factor underlying the decreased CMAP in ICU patients with AQM. This is further supported by the restoration of the denervation induced change in muscle resting potential by muscle activity [22].

Muscle contraction is severely deregulated in ICU patients with AQM [4], [5] due to an imbalance between the rate of protein synthesis and degradation leading to a general myofibrillar protein loss, a preferential loss of myosin and myosin-associated thick filament proteins, muscle fibre atrophy and decreased force [4]. In none of the groups included in this study were there any signs of the preferential myosin loss considered to be a pathognomonic finding in ICU patients with AQM [4]. This is probably due to the relatively short duration of the experimental five-day period. This is supported by recent findings from our group using a rodent ICU model where animals were mechanically ventilated, sedated and exposed to NMBA for durations varying between six hours and two weeks. Similar results were observed in this group as in the MV and NMBA groups in this study, i.e., there was no significant change in maximum single muscle fibre force, myosin∶actin ratio or cross-sectional area during the first four days. However, at durations longer than a week there was a progressive decline in specific force and fibre size in parallel with a preferential myosin loss (submitted manuscript). The dramatic decline in specific force in animals exposed to sepsis or systemic steroid hormone treatment during the five-day period without a preferential myosin loss show that both sepsis and CS augment the effects of the ICU intervention on skeletal muscle by impairing muscle function via mechanisms distinct from altered muscle membrane properties or a preferential myosin loss. This further supports the notion that neither CS nor sepsis represents prerequisites for AQM, but that they facilitate the development of this neuromuscular disorder.

Both sepsis and CS are commonly associated with an increase in circulating glucocorticoid levels [23]. Glucocorticoids are known to exacerbate the deleterious effects of immobilization on muscle structure and function [24]. Different intracellular mediators are involved including Akt, mTOR, GSK-3β, β-catenin, FOXO, REDD1 and ATF4 [25], [26] and provoke a decrease in both the rate of myofibrillar protein synthesis and degradation [25], [26], [27]. Thus, sepsis and CS may induce an early general loss of contractile proteins, preceding atrophy and preferential myosin loss, resulting in a decrease in specific force (Figure 2). In addition, increasing circulating glucocorticoid levels induce an increase in the number of reactive oxygen species [28] and oxidative stress mediated via systemic corticosteroid hormone treatment and sepsis may accordingly lead to post-translational modifications of contractile proteins [29] with negative effects on protein function and decreased specific force.

In conclusion, the present results show that different factors trigger the decreased amplitude of the compound muscle action potential and the decreased force generating capacity of skeletal muscle in response to five days exposure to different experimental ICU conditions. Immobilization in combination with sedation and mechanical ventilation appear to be the key elements triggering the lowering of the CMAP amplitude, probably via an effect on muscle membrane excitability. Sepsis and systemic corticosteroid hormone treatment, separately or in combination, decreased the force generation capacity at the single muscle fibre level, independent of muscle fibre type. However, muscle fibre size was not affected by mechanical ventilation, immobilization, sedation, neuromuscular blocking agents, sepsis or corticosteroids during the five-day experimental period. Thus, the unique porcine ICU model gives valuable insights into the mechanisms underlying limb muscle weakness during the early phase of the ICU intervention and is forwarded as a valuable model for further mechanistic studies as well as in specific intervention studies.
